# High-Throughput Molecular Characterization of the Microbiome in Breast Implant-Associated Anaplastic Large Cell Lymphoma and Peri-Implant Benign Seromas

**DOI:** 10.3390/cancers17233839

**Published:** 2025-11-29

**Authors:** Evelina Rogges, Giorgio Bertolazzi, Davide Vacca, Marina Borro, Gianluca Lopez, Maurizio Simmaco, Anna Scattone, Guido Firmani, Michail Sorotos, Fabio Santanelli di Pompeo, Niccolò Noccioli, Emanuele Savino, Andrea Vecchione, Arianna Di Napoli

**Affiliations:** 1 Department of Medical and Surgical Sciences and Translational Medicine, PhD School in Translational Medicine and Oncology, Sapienza University of Rome, Faculty of Medicine and Psychology, 00189 Rome, Italy; evelina.rogges@uniroma1.it; 2 Pathology Unit, Department of Clinical and Molecular Medicine, Sant’Andrea University Hospital, Sapienza University of Rome, 00189 Rome, Italy; andrea.vecchione@uniroma1.it; 3 Department of Medicine and Surgery, Kore University of Enna, 94100 Enna, Italy; giorgio.bertolazzi@unikore.it; 4 Tumor Immunology Unit, Department of Health Sciences, University of Palermo, 90127 Palermo, Italy; davide.vacca@outlook.com; 5 Center for Precision Medicine (CPM), Sant’Andrea University Hospital, 00189 Rome, Italy; marina.borro@uniroma1.it (M.B.); maurizio.simmaco@uniroma1.it (M.S.); 6 Department of Neuroscience, Mental Health and Sensory Organs (NESMOS), Faculty of Medicine and Psychology, Sapienza University of Rome, 00189 Rome, Italy; 7 Unit of Laboratory Analysis and Advanced Molecular Diagnostics, Department of Diagnostic Sciences, Sant’Andrea University Hospital, 00189 Rome, Italy; 8 Morphologic and Molecular Pathology Unit, St. Andrea University Hospital, Sapienza University of Rome, 00189 Rome, Italy; gianluca.lopez10@gmail.com (G.L.); nocciolin@gmail.com (N.N.); 9 Department of Pathology, IRCCS Istituto Tumori ‘Giovanni Paolo II’, 70124 Bari, Italy; a.scattone@oncologico.bari.it; 10 Plastic Surgery Unit, Department of Neuroscience, Mental Health, and Sense Organs (NESMOS), Faculty of Medicine and Psychology, Sapienza University of Rome, 00189 Rome, Italy; guido.firmani@uniroma1.it (G.F.); michail.sorotos@uniroma1.it (M.S.); fabio.santanelli@uniroma1.it (F.S.d.P.); 11 Advanced Pathology Laboratory, IFOM ETS-The AIRC Institute of Molecular Oncology, 20139 Milan, Italy; emanuele.savino@ifom.eu

**Keywords:** BIA-ALCL, microbiome, NGS

## Abstract

**Background**: Breast implant-associated anaplastic large cell lymphoma (BIA-ALCL) is a mature T-cell lymphoma linked to textured breast implants. A leading hypothesis suggests that chronic inflammation, combined with immunological and genetic factors, drives its pathogenesis. Two previous studies investigating bacterial biofilms on breast implant capsules have produced conflicting results, particularly regarding the enrichment of *Ralstonia* spp. **Methods**: We analyzed the microbiota profiles in seroma samples from 10 BIA-ALCL patients and 12 patients with non-neoplastic effusion, subclassified into acute-, mixed-, and chronic-type based on cellular composition. We used two metagenomic approaches: 16S rRNA gene sequencing and Nanopore sequencing with the “What’s in My Pot?” (WIMP) taxonomic classifier. Our analyses included alpha and beta diversity metrics, as well as comparisons of Gram status and oxygen requirements. **Results**: Both sequencing methods identified *Staphylococcaceae*, *Propionibacteriaceae*, and *Bradyrhizobiaceae* as the most prevalent bacterial families in both BIA-ALCL and benign seroma samples. Notably, the *Burkholderiaceae* family was more abundant in some of the benign seromas according to the 16S rRNA sequencing, but *Ralstonia* spp. were not detected. BIA-ALCL showed higher richness (based on Nanopore data) and higher evenness (based on 16S rRNA data) compared to acute-type seromas, indicating a more homogenous representation of the different taxa identified. BIA-ALCL seromas did not cluster together based on Nanopore data, but they did form a distinct cluster with 16S rRNA data. This cluster was differentiated from the other two clusters by a relatively balanced presence of multiple families without overt dominance. We observed no significant differences in Gram staining between BIA-ALCL and benign samples using either method. However, non-aerobic bacterial families were enriched in BIA-ALCL cases only when analyzed with the Nanopore pipeline. **Conclusions**: Overall, our findings did not identify a distinctive microbial signature specifically associated with BIA-ALCL.

## Simple Summary

Breast implant-associated anaplastic large cell lymphoma (BIA-ALCL) is a rare type of T-cell lymphoma linked to textured breast implants. A leading hypothesis suggests that chronic inflammation, possibly triggered by bacteria forming biofilms on the implant surface, may play a role in its development. However, previous studies based on breast implant capsules have yielded inconsistent results. In our study, we investigated the microbiota in late-onset seromas from 10 BIA-ALCL patients and 12 with a non-neoplastic effusion. Using two different next-generation sequencing methods, we found no single bacterial species or family specifically associated with BIA-ALCL. Instead, BIA-ALCL samples showed a higher abundance of non-aerobic bacteria, which aligns with prior evidence of a hypoxic (low-oxygen) tumor environment. Our findings suggest that while bacteria may foster chronic inflammation, BIA-ALCL likely stems from a combination of factors, rather than a specific infectious cause.

## 1. Introduction

Breast implant-associated anaplastic large cell lymphoma (BIA-ALCL) is an uncommon mature T-cell lymphoma that arises in relation to a textured breast implant [1]. This CD30-positive, anaplastic lymphoma kinase (ALK)-negative malignancy has been increasingly recognized, with the Food and Drug Administration (FDA) reporting a total of 1380 cases of BIA-ALCL with 64 known deaths as of 8 July 2025 [2]. The initial sign of disease is typically a late-onset peri-implant seroma, whereas tumor mass or regional lymphadenopathy are less frequent presentations. Diagnostic protocols for aspirated fluid involve cultural analysis, cytomorphological examination, immunocytochemistry for CD30 expression, and eventual T-cell receptor gene rearrangement analysis [3,4]. However, most late seromas are benign, often linked to infection, trauma, or implant rupture. Based on the cellular composition, we previously sub-classified these late-onset non-neoplastic seromas as acute-type (>50% neutrophils), mixed-type (5 to 50% neutrophils admixed with monocytes and lymphocytes), and chronic-type (when monocytes or lymphocytes predominate) [3].

To date, the etiopathogenesis of BIA-ALCL remains unclear but is considered multifactorial. A prominent hypothesis suggests that in genetically predisposed individuals, implant-associated compounds together with bacterial biofilm on the implant surface may chronically stimulate immune cells in hypoxic conditions [5,6]. This persistent stimulation can lead to proliferating T-cells accumulating genomic alterations, eventually transforming into lymphoma cells. This hypothesis is further sustained by genomic studies identifying somatic genetic alterations of genes involved in the JAK/STAT3 inflammatory signaling, TP53, or in epigenetic modifiers [7,8,9,10,11,12,13]. The constitutive activation of the JAK/STAT3 inflammatory pathway is also consistently evidenced by the expression of phosphorylated STAT3 and transcriptional activation of its target genes [9,12,14,15].

According to the most recent edition of the International Organization for Standardization (ISO) 14607:2024 [16], mammary implant surfaces are classified as either smooth or intentionally textured. Smooth surfaces have a surface roughness (Ra) of 0 μm, while intentionally textured surfaces are further subdivided into microtextured (0 μm < Ra < 50 μm) and macrotextured (Ra ≥ 50 μm) [16]. Recently, a moderate level of evidence linked the development of BIA-ALCL to textured implants, particularly in relation to implants with an intermediate to high surface roughness [17] (Scientific Committee on Health, Environmental and Emerging Risks, SCHEER 2021), primarily due to the higher global incidence of BIA-ALCL cases reported in patients with such devices [1,2]. Highly textured implants have also been associated with increased macrophage activity, scar tissue formation, higher levels of inflammatory T cells [18,19,20], and greater bacterial colonization [21,22,23,24], all contributing to chronic inflammation.

Regarding the bacterial biofilm, Gram-positive bacteria such as *Staphylococcus aureus* and *Staphylococcus epidermidis* are established pathogens in breast implant infections [25]. Conversely, Gram-negative bacterial lipopolysaccharide (LPS) has shown greater efficacy in triggering the activation and proliferation of BIA-ALCL tumor cells in vitro compared to Staphylococcal superantigen enterotoxin A (SEA) [26]. Previous studies on the microbiome of implant capsules have yielded conflicting results. Hu et al., by pyrosequencing the V1–V3 region of the 16S rRNA gene, reported a significantly greater proportion of the Gram-negative *Ralstonia* spp. in 19 BIA-ALCL capsular specimens compared with 12 nontumoral capsules, where a higher number of *Staphylococcus* spp. were found [27]. In contrast, Walker et al. found no significant differences in the relative abundance of Gram-negative bacteria between seven BIA-ALCL capsules and control specimens (i.e., implants, contralateral breast, skin, and one seroma). They also detected low abundance of *Ralstonia* spp. in their samples, attributing this discrepancy to different sequencing methods (next generation sequencing (NGS) vs. pyrosequencing) of the V1–V3 region of 16S rRNA gene [28]. This controversy leaves open the discussion on the precise role of bacterial biofilm in BIA-ALCL pathogenesis. In the current study, we profile for the first time the microbiota of the effusions in a cohort of patients with both neoplastic and benign seromas by employing two distinct metagenomics methods: the 16S rRNA gene NGS and the Nanopore sequencing, a third-generation NGS, coupled with the “What’s in my Pot?” (WIMP) data analysis pipeline. We focused on fresh-frozen seroma fluid, as the seroma represents the primary biological microenvironment in which BIA-ALCL develops. Seroma fluid captures the active peri-implant niche more directly than capsular tissue and provides a highly biologically relevant substrate for microbiome characterization. Indeed, fresh-frozen seroma samples not only preserve microbial DNA with minimal degradation but also avoid the artifacts introduced by the processing and storage of formalin-fixed and paraffin-embedded (FFPE) capsule tissue.

## 2. Materials and Methods

### 2.1. Sample Collection

Twenty-two late-onset fresh seroma samples from 22 patients with a diagnosis of BIA-ALCL (10 samples) or benign effusion (12 samples) were collected at Sant’Andrea University Hospital of Rome, Italy, from 2014 to 2020. Patients with non-neoplastic effusions were further subclassified into three types according to their cellular composition as previously described: acute-type (4 samples), mixed-type (3 samples), and chronic-type (5 samples) [3]. All patients were women and had textured implants. Patients with non-neoplastic effusions had a mean age of 53 years (range 33–69), and 7 out of 12 (58.3%) had a history of breast cancer. For patients diagnosed as BIA-ALCL, the mean age was 55 years (range 38 to 76), and their implants were placed for either reconstructive (6 out of 10, 60%) or cosmetic reasons (4 out of 10, 40%). The BIA-ALCL cases were staged according to the MD Anderson TNM staging system [29] as follows: stage IA (6 patients), stage IB (1 patient), stage IIA (2 patients), and stage IIB (1 patient). The clinical characteristics of both the benign and BIA-ALCL samples, along with the pathological stages for BIA-ALCL patients, are detailed in Table 1 and Table 2. All fresh seroma samples were immediately frozen at −80 °C until DNA extraction. DNA was extracted using the QIAamp ^®^ DNA Mini Kit (Qiagen, Hilden, Germany) and quantification was assessed using Qubit™ dsDNA Quantification High Sensitivity Assay Kits (ThermoFisher Scientific, Waltham, MA, USA). Two distinct DNA aliquots were taken for Nanopore and 16S rRNA gene sequencing.

### 2.2. Nanopore Sequencing and Data Analysis

The libraries were prepared on 100 ng input DNA extracted from each seroma by using the polymerase chain reaction (PCR) barcoding protocols (EXP-SQK-PBK004, Version: PBK_9073_v1_revM) (Oxford Nanopore Technologies (ONT), Oxford, UK). Sequencing was performed on an ONT MinION flow cell (FLO-MIN106 R9 Version) connected to a Mk1B device (ONT Ltd.; MIN-101B, Oxford Nanopore Technologies, Oxford, UK) following the manufacturer’s instructions [30]. The sequencing was run up to 48 h each time, using Nanopore software MinKNOW v3.1.19 (for the first and second runs) and MinKNOW v4.0.4 for the last run (Oxford Nanopore Technologies Ltd., UK), which collects the sequencing data and converts it into base-called reads. For the post-base-calling analysis, the cloud-based EPI2ME software v.3.4.2.1259 [31] (https://epi2me.nanoporetech.com/about/ (accessed on 15 March 2019, 20 March 2019, and 26 August 2020) with “What’s in my pot?” (WIMP) (quantitative analysis tool for real-time species identification—a part of EPI2ME) was used for the evaluation of species diversity. WIMP filters fastq files with a mean q-score below a minimal threshold and the centrifuge classification engine assigns each read to a taxon in the National Center for Biotechnology Information (NCBI) taxonomy and reference database [32,33,34].

This method covers bacterial, viral, and fungal genomes [34,35]. We used the taxname function from the *taxize* R package (R version 4.5.0) [36] to retrieve family, genus, and species names from the NCBI database and assign them to the organisms identified by WIMP; patients were divided into three runs composed of 4, 8, and 10 DNA samples each (Supplementary Figure S1). In each run, a control sample extracted from a Human Herpesvirus 8 (HHV8)-positive primary effusion lymphoma (PEL) was added, and the nuclease-free water was used as a blank test. All fastq files are available on request to the corresponding author. We did not observe any batch effect among the samples from different runs (Supplementary Figure S1). Indeed, the clusters derived from the beta diversity analysis using the Bray–Curtis matrix were formed independently of the sequencing run.

### 2.3. Microbiome Sequencing by 16S rRNA Gene Sequencing and Data Analysis

The Ion16S™ Metagenomics Kit (ThermoFisher Scientific, Waltham, MA, USA) was used to obtain the library from the DNA extracted from 21 samples. The DNA of patient Chronic-05 was excluded as it was not sufficient for the analysis. The kit includes two sets of primers to amplify the V2, 4, 8 and the V3, 5, 6, 7, 9 hypervariable regions of the bacterial 16S rRNA gene. Template preparation and chip loading were carried out using an IonS5 Ion Chef™ System (ThermoFisher Scientific, Waltham, MA, USA), and sequencing was performed using an IonS5 System (ThermoFisher Scientific, Waltham, MA, USA). After sequencing, the fastq files were processed using a custom script based on the Quantitative Insights Into Microbial Ecology 2 (QIIME2) software (version QIIME2 2023.5). Quality control retained sequences with a length between 140 and 400 base pairs (bps) and a mean sequence quality score > 20, while sequences with homopolymers > 7 bps and mismatched primers were omitted. To calculate downstream diversity measures, 16SrRNA Operational Taxonomic Units (OTUs) were defined at 100% sequence homology; OTUs not encompassing at least 2 sequences of the same sample were removed. All reads were classified to the lowest possible taxonomic rank using QIIME2 software and a reference dataset from the Curated MicroSEQ(R) 16S Reference Library v2013.1 (ThermoFisher Scientific) and Curated Greengenes v13.5 [37]. Fastq files are available on request to the corresponding author.

### 2.4. Statistical Analysis

For hierarchical clustering analysis of the samples, the Bray–Curtis distance metric was considered, and the Ward-D2 aggregation method was used for building the dendrogram within the R package hclust. The relative counts were considered for the bar plot representation. The microbiome differential abundance analysis between BIA-ALCL and non-BIA-ALCL groups was performed by applying the ancombc2 function of the Analysis of Compositions of Microbiomes with Bias Correction (ANCOMBC) R package [38] to the original count matrix. The diversity indices were calculated in R based on their formula [39,40]. Fractions and indices were compared between the BIA-ALCL and non-BIA-ALCL groups through the non-parametric bootstrap t-test. All statistical analyses have been performed using R statistical software v4.5.0 [41].

## 3. Results

### 3.1. Abundance Analysis of BIA-ALCL and Benign Samples

Using Nanopore’s WIMP workflow, we identified a total of 85 unique bacterial, 4 viral, and 6 fungal families across all samples. Specifically, 55 bacterial, 4 viral, and 4 fungal families were detected in BIA-ALCL samples, while benign seromas contained 56 bacterial, 6 fungal, and 3 viral families (Table 3 and Supplementary Table S1). In contrast, 16S rRNA sequencing, which targets only bacterial genomes, identified a total of 222 bacterial families. Of these, 130 were found in BIA-ALCL samples and 212 in benign seromas (Table 4 and Supplementary Table S1).

**Figure 1:**
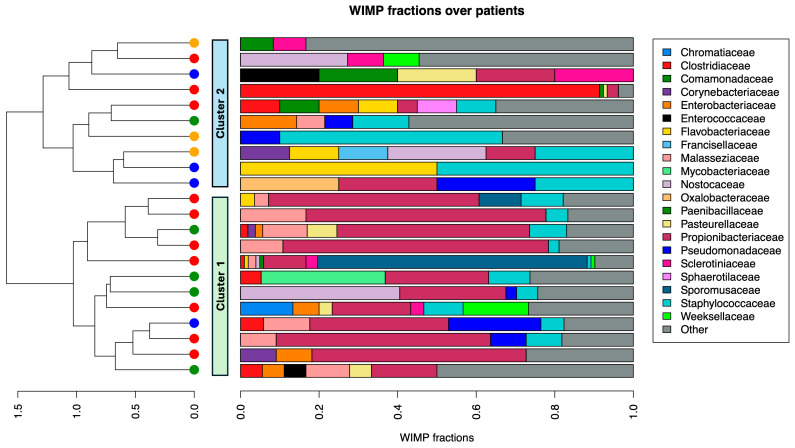

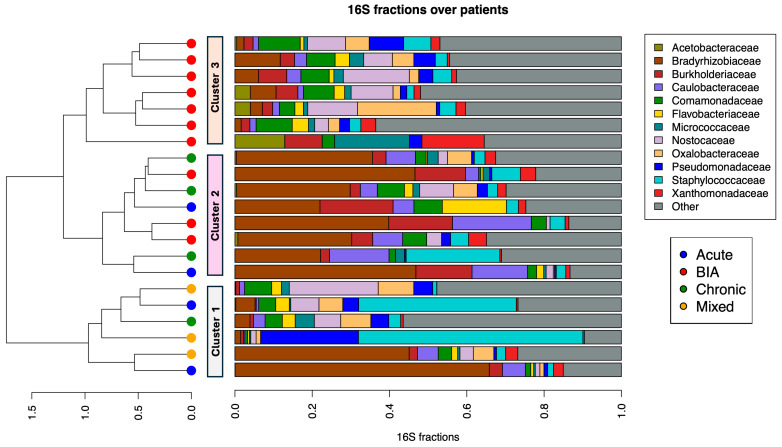
Relative frequencies of organism families across patients, ordered by an unsupervised cluster analysis based on the Bray–Curtis dissimilarity matrix. Using Nanopore sequencing data, samples were segregated into two clusters, independent of the type of effusion (**upper** panel). In contrast, using the 16S rRNA sequencing data, most BIA-ALCL samples clustered together in cluster 3 (**lower** panel).

Based on Nanopore sequencing, the most represented families, in both BIA-ALCL and non-neoplastic seromas, were *Propionibacteriaceae and Staphylococcaceae* (Figure 1, upper panel and Supplementary Table S2). Conversely, 16S rRNA gene sequencing revealed that *Bradyrhizobiaceae* and *Staphylococcaceae* predominated (Figure 1, lower panel and Supplementary Table S2).

We then calculated the beta diversity to examine differences in the types and proportions of microbes present across different samples, using the unsupervised Bray–Curtis dissimilarity matrix (Figure 1). Nanopore sequencing showed no significant difference between benign and BIA-ALCL seromas. However, patients, regardless of the type of seromas, clustered in two distinct groups (Figure 1, upper panel). The major families driving the diversity between these two groups were *Propionibacteriaceae* (*p* < 0.001), *Malasseziaceae* (*p* = 0.006), *Pasteurellaceae* (*p* = 0.009), and *Sclerotiniaceae* (*p* = 0.02), all of which were more represented in cluster 1.

Using 16S rRNA sequencing data, 7 out of 10 BIA-ALCL seromas clustered together (cluster 3) (Figure 1, lower panel). Unlike clusters 1 and 2, where single taxa often dominated the community, samples in cluster 3 shared a relatively balanced presence of multiple families without overt dominance. Notably, numerous families were significantly underrepresented in cluster 3, including *Burkholderiaceae* (log fold change (logFC) = −1.235, *p* = 0.005), which encompasses *Ralstonia* spp. that were not detected.

Due to the overall low abundance and impact of viruses and fungi on the clustering of samples, our further analyses on Nanopore data focused solely on bacterial families. We then investigated whether any microbial families were recurrently associated with BIA-ALCL or benign seroma patients by performing a Fisher’s exact test on each family, based on its presence or absence across samples. Using both sequencing approaches, we found no microbial family significantly enriched in the BIA-ALCL group (adjusted *p*-values > 0.05, Supplementary Table S3).

To gain a deeper understanding of the microbiome characteristics within the BIA-ALCL environment, we performed a differential abundance analysis comparing the microbiomes of BIA-ALCL and benign groups (see Methods Section). Only a limited number of bacterial families were found to be differentially abundant between the two conditions (Supplementary Table S4). Specifically, just one family (*Clostridiaceae*) showed higher abundance in the BIA-ALCL group based on Nanopore sequencing. However, this trend was not consistent across sequencing methods. Indeed, in the 16S rRNA dataset, *Clostridiaceae* exhibited greater abundance in benign samples. The 16S analysis identified a greater number of differentially abundant families (log2FC > 0.58, adjusted *p*-value < 0.05; see Supplementary Table S4). Nonetheless, all these families displayed a low average relative abundance (<10%) in the BIA-ALCL group and were also present in the benign seroma group (Supplementary Table S2). These findings suggest that no specific bacterial families are uniquely or strongly enriched in the BIA-ALCL microbiome.

**Figure 2:**
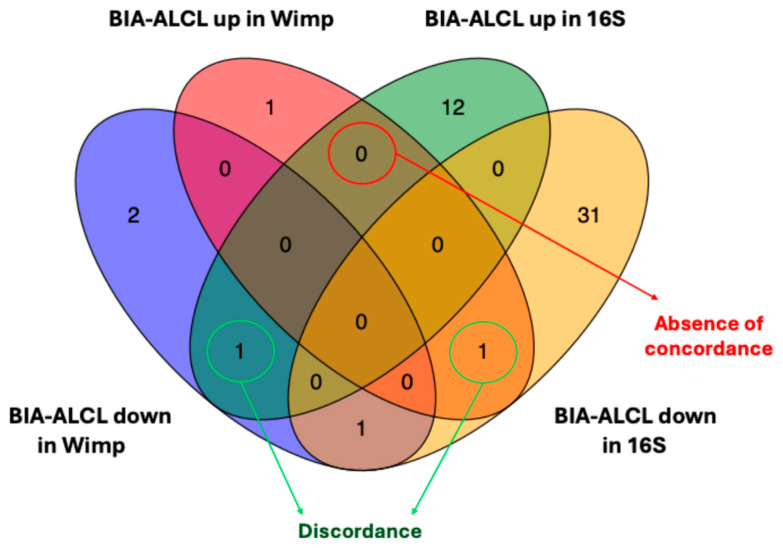
Venn diagram showing the overlap of microbial families identified as differentially abundant in BIA-ALCL patients across two sequencing methods (WIMP and 16S rRNA, adjusted *p*-values < 0.1). Each section represents families that were either increased (“BIA-up”) or decreased (“BIA-down”) in the BIA-ALCL group, as determined by ANCOM-BC. The absence of overlap among methods indicates a lack of concordance in the identified differentially abundant families. The central area with no shared families highlights the non-concordant results (red circle).

To assess whether the abundance differences showed a similar pattern between the outputs of WIMP and 16S, we selected a broader group of families that show a differential abundance behavior (adjusted *p*-value < 0.1, no threshold on log_2_FC). Then we evaluated the concordance between the two sets of identified families. As shown in the Venn diagram (Figure 2), no families are displaying a consistent trend across the two sequencing methods. This result is consistent with the lack of a predominant family, as indicated by the results of Fisher’s exact test (Supplementary Table S3). In our view, what we have observed is likely due to the absence of families with a marked difference in abundance that could truly characterize the BIA-ALCL patient group.

### 3.2. Bacterial Family Differences Within Samples by Using Alpha Diversity Assays

To uncover eventual differences within each benign and BIA-ALCL group, we calculated alpha diversity, which provides information on the diversity of the microbial community within individual samples by quantifying the richness (number of different microbial species) and the evenness (distribution of those species). Alpha diversity was assessed using richness alone (absolute number of distinct families) or both richness and evenness (Shannon index and inverse Simpson index).

**Figure 3:**
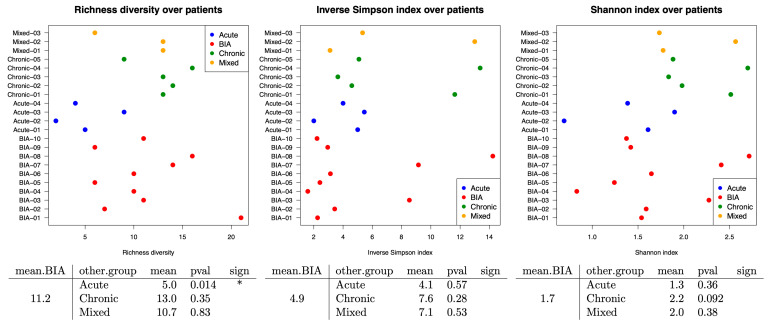
Alpha diversity, measured at the family level using Nanopore sequencing data, was compared across patient groups. BIA-ALCL had a significantly (<0.05 *) higher median value of richness when compared to acute-type seromas (*p* = 0.014).

Based on Nanopore sequencing, BIA-ALCL samples did not show any significant differences when compared to all benign seromas. However, when specifically compared to benign acute-type seromas, BIA-ALCL had a significantly higher median value of richness (*p* = 0.014) (Figure 3), suggesting BIA-ALCL had a higher number of different bacterial families.

**Figure 4:**
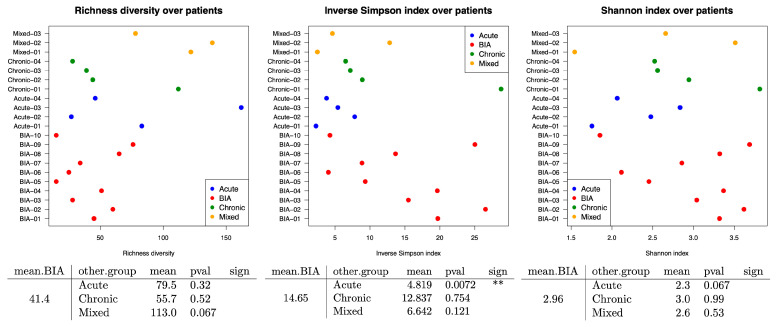
Alpha diversity, measured at the family level using 16S rRNA gene sequencing data, was compared across patient groups. The inverse Simpson index revealed significantly (<0.01 **) higher microbial diversity in BIA-ALCL samples compared to the benign acute-type group (*p* = 0.0072).

Moreover, the inverse Simpson index evaluated on 16S rRNA gene sequencing data (Figure 4) revealed that the BIA-ALCL group had significantly higher microbial diversity than the benign acute-type group (*p* = 0.0072). This difference suggests a loss of evenness, with the dominance of a few taxa, during acute infection. Conversely, BIA-ALCL seromas host more compositionally diverse communities, which is more consistent with a chronic inflammatory process.

### 3.3. Impact of Bacteria GRAM Stain in Benign and BIA-ALCL Seromas

**Figure 5:**
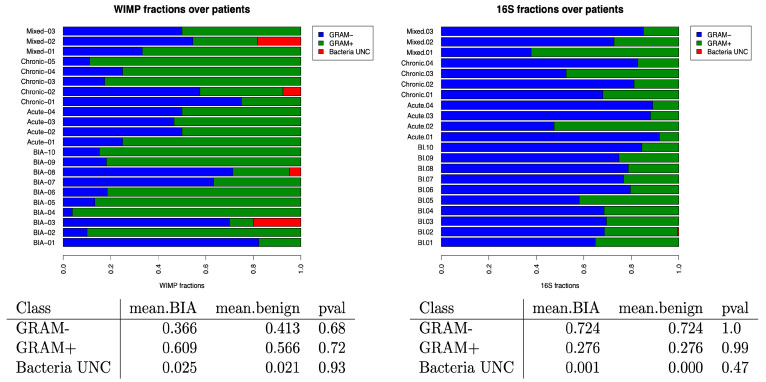
Relative frequencies of Gram-positive (GP, green bars) and Gram-negative (GN, blue bars) bacteria across patients with BIA-ALCL and benign seromas. No statistically significant differences were found in the proportion of GP and GN bacteria between BIA-ALCL and benign samples using either sequencing method. Red bars indicate the non-classified bacteria.

We successfully classified most bacteria in our cohort (99%) as either Gram-positive (GP) or Gram-negative (GN). This classification relied on three different databases: BacDive [42], UK Culture Collections [43], and BactoTraits [44]. Upon assessing their distribution across samples and using both sequencing datasets, we found no significant differences in the proportion of GP and GN between BIA-ALCL and benign seromas (Figure 5).

### 3.4. Oxygen Tolerance of the Bacteria in Benign and BIA-ALCL Seromas

**Figure 6:**
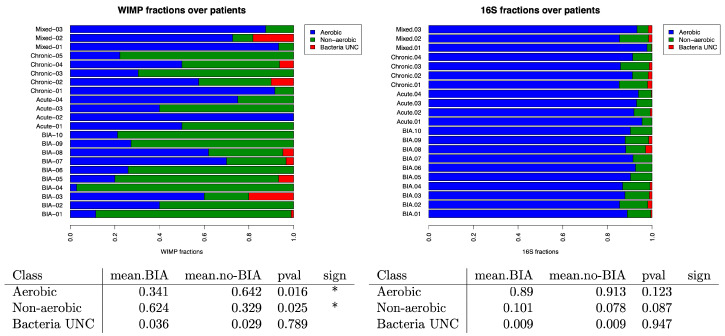
Relative frequencies of aerobic and anaerobic bacteria compared across patients with BIA-ALCL and benign seromas. BIA-ALCL samples were significantly (<0.05 *) enriched in non-aerobic bacterial families, but this was statistically significant only with WIMP data.

Considering the known hypoxic microenvironment of peri-implant seromas [6], we classified the bacteria according to their oxygen requirements using the same three databases: BacDive [42], UK Culture Collections [43], and BactoTraits [44]. We were able to classify most of the bacteria of our cohort (95.4%) based on their oxygen tolerance. Nanopore data revealed a significant enrichment of non-aerobic (anaerobic and microaerophilic) bacteria in BIA-ALCL samples compared to benign ones (*p* = 0.025). Although 16S rRNA sequencing data showed a trend towards more non-aerobic bacteria in BIA-ALCL compared to the benign seromas, this difference did not reach statistical significance (*p* = 0.087) (Figure 6).

## 4. Discussion

This study provides the first comprehensive characterization of the microbiota in late-onset textured breast implant seromas, encompassing both BIA-ALCL and non-neoplastic effusions, by utilizing seroma fluid samples and a dual sequencing approach: Nanopore sequencing with the WIMP workflow and 16S rRNA gene sequencing. Both methods failed in identifying a microbiome profile specifically associated with BIA-ALCL.

Previous studies on the BIA-ALCL bacterial community have focused more on breast implant capsules than seroma samples; however, they present conflicting perspectives. Both Walker et al. and Hu et al. employed 16S rRNA gene sequencing (V1–V3 regions) with the same taxonomic classifier but different sequencing methods (Illumina NGS vs. FLX pyrosequencing) [27,28]. Hu et al. identified *Ralstonia* spp. as a major driver of diversity in BIA-ALCL [27], while Walker et al. found no consistent differences and low abundance of *Ralstonia* spp., primarily in non-ALCL controls [28]. Walker et al. also identified *Staphylococcaceae* and *Propinobacteriaceae* as common colonizers of the breast environment rather than disease-specific pathogens. Our findings corroborate those reported by Walker et al. Regardless of the sequencing method applied, *Staphylococcaceae*, *Propionibacteriaceae*, and *Bradyrhizobiaceae* emerged as among the most prevalent bacterial families in both BIA-ALCL and benign seroma samples. In addition, *Burkholderiaceae*—the family encompassing *Ralstonia* spp.—was detected predominantly in non-BIA-ALCL samples, with no evidence of *Ralstonia* spp. themselves.

We also showed the presence of higher richness and evenness in BIA-ALCL compared with acute seromas. This observation supports a biological model in which BIA-ALCL arises within a chronic inflammatory environment rather than an acute infectious setting. Specifically, acute seromas typically show low evenness due to the overgrowth of a single pathogen, whereas BIA-ALCL displays a more ecologically balanced microbial structure, consistent with chronicity.

In general, Nanopore sequencing detected a lower number of bacteria families when compared to 16S rRNA sequencing, potentially due to the method’s lack of an amplification step. Nevertheless, our positive control (PEL) showed a high number of HHV8 reads, confirming the detection of a known disease-driving pathogen. Furthermore, *Pseudomonas aeruginosa* was successfully cultured from one benign mixed-type seroma in a previous study [3] and consistently confirmed by both WIMP and 16S rRNA sequencing in the present study. These observations suggest that disease-defining organisms tend to be more abundant and readily detected, implying that a single microorganism is unlikely to be solely responsible for BIA-ALCL pathogenesis.

While beta diversity analysis with the WIMP workflow failed to distinguish BIA-ALCL from benign effusions, the 16S rRNA sequencing data revealed a tendency for BIA-ALCL samples to form a more distinct cluster (Figure 1 cluster 3). This cluster, notably, exhibited a highly even community composition, differentiating it from the other two clusters, where individual taxa dominated. Indeed, cluster 3 does not reflect the presence of specific pathogens or a unique taxonomic signature. Instead, this clustering pattern arises from a compositional profile that is distinct (more even) but not dominated by any particular taxon. As shown by the enrichment analysis (Supplementary Table S3), while non-neoplastic seromas were significantly enriched in several taxa, no taxa showed significant enrichment in the BIA-ALCL group. In keeping with this result, BIA-ALCL samples showed higher richness (based on Nanopore data) and a higher evenness (based on 16S rRNA data) compared to acute-type seromas, indicative of a more homogenous representation of the different taxa identified. This pattern of community structure further supports the hypothesis that the singular overgrowth of a specific pathogen does not characterize BIA-ALCL.

Mempin et al. highlighted a potential role for Gram-negative bacteria in BIA-ALCL pathobiology, observing that BIA-ALCL tumor cells responded more to LPS stimulation in vitro compared to other mitogenic agents [26]. In contrast, our study found no statistically significant differences in the abundance of Gram-negative and Gram-positive bacteria in BIA-ALCL when compared to benign seromas, a result also corroborated by Walker et al. [28].

Intriguingly, Oishi et al.’s gene expression profiling of BIA-ALCL identified an upregulation of hypoxia signaling genes, including carbonic anhydrase-9 (CA9), which can stimulate BIA-ALCL cell lines in vitro [6]. Consistent with this, our Nanopore data showed anaerobic and microaerophilic bacteria were significantly more abundant in BIA-ALCL, with 16S rRNA sequencing showing a similar trend. The enrichment of non-aerobic bacteria observed in BIA-ALCL seromas—significant in Nanopore data and trending in 16S—should, however, be interpreted cautiously. This association is correlative and does not establish a causal link between the hypoxic tumor microenvironment and microbial composition. Rather, it may reflect ecological compatibility with the biochemical milieu of peri-implant seromas, without implying selective microbial participation in lymphomagenesis. Establishing a true causal role for anaerobic bacteria in BIA-ALCL would require functional assays and in-depth biological investigations that could also provide additional insights into microbial metabolism and potential virulence. As our primary goal was to clarify whether the disease is associated with a dominant pathogen or rather with a diverse microbial milieu, functional predictions were beyond the scope of this study and should be pursued in future research with larger and functionally oriented datasets.

A limitation of our study was the lack of direct concordance between Nanopore-based metagenomics (using the WIMP workflow) and 16S rRNA sequencing at the individual sample level. This discrepancy is attributable mainly to inherent technical differences in sample processing, sequencing targets, and bioinformatics. Shotgun sequencing offers broad detection of DNA but has lower sensitivity for low-abundance taxa due to a lack of amplification [45]. Conversely, while 16S rRNA sequencing is more focused and sensitive for bacterial detection, it can introduce amplification biases. Consequently, taxonomic profiles generated by each method can differ significantly per sample. Additionally, the different classification databases employed (NCBI RefSeq for WIMP vs. Greengenes/MicroSEQ for 16S rRNA) may further contribute to these discrepancies. The WIMP workflow performs taxonomic assignment by aligning raw reads against the comprehensive NCBI RefSeq database. While this broad genomic reference enables species-level resolution when complete genomes are available, it may underperform when target organisms are underrepresented or poorly annotated in the RefSeq database. In contrast, 16S rRNA gene sequencing relies on amplification of specific hypervariable regions of the 16S rRNA gene, with taxonomic classification based on curated reference libraries such as Greengenes or MicroSEQ [45]. Classification rate and accuracy and taxonomic read classification have been reported to be heavily impacted by the reference database choice, with NCBI RefSeq showing a lower performance when compared to other databases, particularly at low taxonomic ranks [46,47]. These methodological and database differences likely explain why in our work certain families were differentially represented across the two methods and why some were exclusively detected by one method. Despite these individual sample variations, both platforms successfully captured overlapping microbial trends at the group level. This demonstrates the value of multi-modal sequencing for investigating complex microbiota in breast implant-associated effusions and confirms that BIA-ALCL is associated with complex ecological patterns rather than a single pathogen.

The lack of concordance between Nanopore-based metagenomics and 16S rRNA amplicon sequencing precludes any direct, sample-level taxonomic comparison between the two platforms. This discrepancy is expected, as shotgun sequencing and amplicon-based approaches differ substantially in sensitivity, amplification biases, and reference database structure. An example is *Clostridiaceae*, a family found more abundant in the BIA-ALCL group based on Nanopore sequencing and in benign samples by 16S rRNA sequencing. Despite this methodological discordance, both techniques converged on the same overarching conclusion: BIA-ALCL seromas do not harbor a reproducible or disease-specific microbial signature. Therefore, the robustness of our findings lies not in taxonomic agreement between methods, but in the consistent group-level pattern observed across two independent sequencing strategies. Of note, the objective of this study was not to characterize the microbiome of each individual patient, but rather to determine whether BIA-ALCL seromas shared a dominant bacterial family at the group level. If such a predominant family were truly present, the two sequencing platforms might classify it differently at the taxonomic level, but it would still emerge as a reproducible signal across samples. Instead, no such dominant family was detected by either method. This indicates that the discordance observed between techniques concerns taxonomic assignment, not the presence or absence of a shared microbial signature. The fact that two technologically distinct approaches both failed to identify a predominant family strengthens the validity of this negative result and is fully consistent with the absence of a disease-specific pathogen previously reported by Walker et al.

Another limitation of our study is the relatively small sample size, which reflects the rarity of BIA-ALCL and the inherent difficulties in assembling larger cohorts even at referral centers. However, this limit is shared with the other two available microbiome studies in BIA-ALCL, which included 26 [27] and 7 [28] BIA-ALCL cases, respectively. Despite this limitation, the consistent negative findings across two independent sequencing platforms we employed provide a solid foundation for our conclusion that no dominant microbial signature characterizes BIA-ALCL seromas.

## 5. Conclusions

In conclusion, our data did not reveal a distinctive microbial signature associated with BIA-ALCL when compared to non-neoplastic seromas, suggesting a lack of direct causality between specific microbial taxa and BIA-ALCL pathogenesis. The development of BIA-ALCL is more plausibly a complex process involving multiple genetic and non-genetic factors, warranting future studies to investigate their interplay.

## Figures and Tables

**Table 1:** Clinical characteristics of the patients with benign effusions.

CASE ID	Type of Seroma	Age	Reason of the Implant
Acute-01	Acute	42	Aesthetic
Acute-02	Acute	43	Reconstruction after breast cancer
Acute-03	Acute	59	Reconstruction after breast cancer
Acute-04	Acute	33	Aesthetic
Mixed-01	Mixed	56	Aesthetic
Mixed-02	Mixed	50	Reconstruction after breast cancer
Mixed-03	Mixed	63	Aesthetic
Chronic-01	Chronic	60	Reconstruction after breast cancer
Chronic-02	Chronic	38	Aesthetic
Chronic-03	Chronic	69	Reconstruction after breast cancer
Chronic-04	Chronic	69	Reconstruction after breast cancer
Chronic-05	Chronic	52	Reconstruction after breast cancer

**Table 2:** Clinical characteristic and pathological stage of the BIA-ALCL patients.

CASE ID	Age (Years)	Reason of the Implant	Time from the Implant (Years)	MD Anderson Stage	Therapy	Outcome
BIA-01	53	Aesthetic	3	IA	Radical en-bloc capsulectomy	CR
BIA-02	67	Reconstruction after breast cancer	13	IIA	Radical en-bloc Capsulectomy+RT	CR
BIA-03	66	Reconstruction after breast cancer	11	IIB	Radical en-bloc capsulectomy+CHOEP	CR
BIA-04	55	Aesthetic	10	IA	Radical en-bloc capsulectomy	CR
BIA-05	49	Reconstruction after breast cancer	8	IA	Radical en-bloc capsulectomy	CR
BIA-06	76	Reconstruction after breast cancer	15	IIA	Radical en-bloc capsulectomy+RT	CR
BIA-07	54	Reconstruction after breast cancer	13	IA	Radical en-bloc capsulectomy	CR
BIA-08	51	Reconstruction after breast cancer	NA	IB	Radical en-bloc capsulectomy	CR
BIA-09	40	Aesthetic	12	IA	Radical en-bloc capsulectomy	CR
BIA-10	38	Aesthetic	6	IA	Radical en-bloc capsulectomy	CR

**Table 3:** Number of microorganism families identified in each seroma group by Nanopore sequencing.

Sample Type	Microorganisms (Total *n*)	Bacterial Families (*n*)	Fungal Families (*n*)	Viral Families (*n*)
BIA-ALCL	63	55	4	4
Benign seroma	65	56	6	3
Acute-type	15	12	2	1
Mixed-type	29	26	2	1
Chronic-type	43	37	4	2

**Table 4:** Number of bacterial families identified in each seroma group by 16S rRNA sequencing.

Sample Type	Bacterial Families (*n*)
BIA-ALCL	130
Benign seroma	212
Acute-type	175
Mixed-type	180
Chronic-type	119

## Data Availability

All data is available upon request to the corresponding author.

## Supplementary Materials

The following supporting information can be downloaded at https://www.mdpi.com/article/10.3390/cancers17233839/s1, Figure S1: Evaluation of batch effect among samples using the Bray–Curtis matrix; Table S1: List of all the organisms identified by both Nanopore and 16S rRNA sequencing; Table S2: Family ranking based on the average fractions over samples; Table S3: Fisher’s exact test for comparing microbial presence/absence between BIA-ALCL and benign groups; Table S4: Differential abundance analysis between BIA-ALCL and benign samples.

## Abbreviations

The following abbreviations are used in this manuscript:

BIA-ALCL	Breast Implant-Associated Anaplastic Large Cell Lymphoma
FDA	Food and Drug Administration
ISO	International Organization for Standardization
SCHEER	Scientific Committee on Health, Emerging and Environmental Risks
NGS	Next Generation Sequencing
WIMP	What’s in my Pot?
ALK	Anaplastic Lymphoma Kinase
HHV8	Herpes Virus 8
QIIME	Quantitative Insights Into Microbial Ecology 2
ANCOMBC	Analysis of Compositions of Microbiomes with Bias Correction
ONT	Oxford Nanopore Technologies
NCBI	National Center for Biotechnology Information
